# Factors influencing general practitioner referral of patients developing end-stage renal failure: a standardised case-analysis study

**DOI:** 10.1186/1472-6963-6-114

**Published:** 2006-09-13

**Authors:** Anthony J Montgomery, Hannah M McGee, William Shannon, John Donohoe

**Affiliations:** 1 Department of Psychology, Royal College of Surgeons in Ireland-Medical University of Bahrain, PO Box 15503, Manama, Bahrain; 2 Health Service Research Centre, Department of Psychology, Royal College of Surgeons in Ireland, 123 St. Stephen's Green, Dublin 2, Ireland; 3 Department of General Practice, Royal College of Surgeons in Ireland, Dublin 2, Ireland; 4 Department of Renal Medicine, Beaumont Hospital, Dublin 9, Ireland

## Abstract

**Background:**

To understand why treatment referral rates for ESRF are lower in Ireland than in other European countries, an investigation of factors influencing general practitioner referral of patients developing ESRF was conducted.

**Method:**

Randomly selected general practitioners (N = 51) were interviewed using 32 standardised written patient scenarios to elicit referral strategies. Main outcome measures: General practitioner referral levels and thresholds for patients developing end-stage renal disease; referral routes (nephrologist vs other physicians); influence of patient age, marital status and co-morbidity on referral.

**Results:**

Referral levels varied widely with the full range of cases (0–32; median = 15) referred by different doctors after consideration of first laboratory results. Less than half (44%) of cases were referred to a nephrologist. Patient age (40 vs 70 years), marital status, co-morbidity (none vs rheumatoid arthritis) and general practitioner prior specialist renal training (yes or no) did not influence referral rates. Many patients were not referred to a specialist at creatinine levels of 129 μmol/l (47% not referred) or 250 μmol/l (45%). While all patients were referred at higher levels (350 and 480 μmol/l), referral to a nephrologist decreased in likelihood as scenarios became more complex; 28% at 129 μmol/l creatinine; 28% at 250 μmol/l; 18% at 350 μmol/l and 14% at 480 μmol/l. Referral levels and routes were not influenced by general practitioner age, sex or practice location. Most general practitioners had little current contact with chronic renal patients (mean number in practice = 0.7, s.d. = 1.3).

**Conclusion:**

The very divergent management patterns identified highlight the need for guidance to general practitioners on appropriate management of this serious condition.

## Background

Historically, the provision of renal replacement therapy for end-stage renal disease in Ireland (57.8 new cases per million population in 1994) [1] has been lower than the internationally recommended rate (80 per million population)[2]. Moreover recent data suggests that while Ireland compares favorably with European averages in regard to Transplant treatment (353 per million population versus EU average 185 per million population), Irish dialysis rates [3] still lag behind the European average [4](303 per million population versus EU average of 400 per million population). This all suggests that Irish treatment rates may indicate under-referral of patients with end-stage renal disease. Increases in treatment rates in the United Kingdom [5] have been achieved by increasing referrals of appropriate cases from primary care services. A number of factors may influence referral rates. In a standardised case study (with co-morbidity held constant), Canadian physicians referred significantly fewer older patients [6]. In a similar study format, British general practitioners referred fewer end-stage renal disease patients when compared with British nephrologists [7]. The issue of under-referral may be compounded by late referral which also represents an important element of the referral process. For example, a survey in Canada, the United States and the UK showed that family physicians referred patients at a serum creatinine of between 260 and 340 mmol/L regardless of patient age, whereas most nephrologists would prefer to accept referrals at lower serum creatinines [8]. For example, the guidelines of the UK Renal Association [9] indicate that all patients with a creatinine between 150–200 mmol/L should be referred to a specialist. Similarly, a European study investigating the optimal time for a first referral to a nephrologist found substantial differences between diabetes experts and nephrologists [10].

Under-referral or late referral has important healthcare management implications. Ultimately, the consequences of late referral of patients includes increased mortality and morbidity [11], increased cost and duration of hospitalization [12], increase need for urgent dialysis [13] and reduced access to renal transplantation services [14]. A recent review of the literature [15] concluded that late referral leads to suboptimal management of complications of chronic renal insufficiency, and thus increased morbidity and mortality of patients on renal replacement therapy.

The aim of this study was to examine decision-making strategies of general practitioners regarding referral for renal replacement therapy in order to provide information on clinical, demographic and service-related factors influencing levels and patterns of referral. Since episodes of patients developing renal failure presenting in general practice are relatively rare, standardised case analysis was used as the method of examining what is likely to happen in actual general practice. Provided that realistic cases are constructed, such written simulations are regarded as suitable for measuring clinical judgements and elucidating the decision making process [16,17]. For instance, standardised case analysis has been demonstrated to be an acceptable method of approximating actual clinical practice for general practitioners (respiratory illness [18] and gastroenteritis [19]) and for specialists (rheumatologists [20]). The main outcomes measures were general practitioner referral levels, thresholds for patients developing end-stage renal disease, referral routes and influence of patient age, marital status and co-morbid condition on referral.

## Method

General practitioners were randomly selected from an urban and a rural setting. The rural setting was selected to represent typical general practice distances from a major renal centre. Members of the Irish College of General Practitioners were invited by letter and follow-up telephone call to take part in an interview study. Following this, each participating GP was visited by the first author who conducted a structured interview using the standardized case developed by the researchers.

A total of 79 general practitioners were randomly identified from area listings. Eight general practitioners were unable to take part as they were on holiday during the research period. A further 6 general practitioners could not be contacted by phone. Of 31 urban general practitioners contacted 25 participated in the study (81% response rate), while 26 of the 27 rural general practitioners participated (96% response rate).

### Interviews comprised two parts

**Figure 1:**
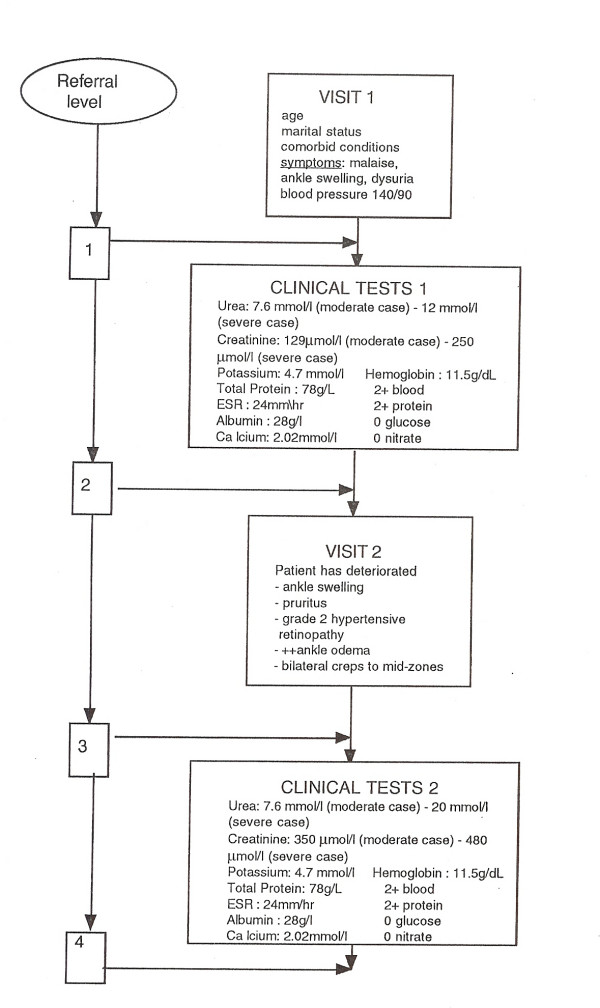
Standardised patient scenario.

a) Case scenarios depicting patients in varying stages of renal failure presenting in general practice. Cases were developed by a general practitioner and nephrologist. They comprised clinical information representing a 'moderate' and 'severe' renal failure profile. Cases were developed as a series of successive general practitioner consultations with laboratory investigation outcomes available where requested. Moderate cases comprised of a patient presenting with symptoms which are associated with relatively low initial creatinine level; 129 μmol/l increasing to 350 μmol/l after a second visit. Equivalent levels in the severe scenarios were 250 μmol/l and 480 μmol/l. Cases also differed in demographic and co-morbidity criteria – age of patient (40 vs 70 years), marital status (single vs married) and co-morbidity (inactive rheumatoid arthritis vs no co-morbidity). This design provided 32 case combinations (see example, fig. 1).

b) Questions on general practitioner demographic profile and training and current experience with renal disease.

## Results

General practitioners (43 men/8 women) averaged 50 years old (s.d. = 9) with a mean practice size of 2.3 (s.d. = 2.3) partners. Practices were located a median of 15 miles (range = 1–65) from the nearest dialysis centre. General practitioners reported little current contact with renal patients; 90% of general practitioners had ≤ 1 hemodialysis, 96% had ≤ 1 continuous ambulatory peritoneal dialysis and 79% had ≤ 1 transplant patients in their practice.

**Figure 2:**
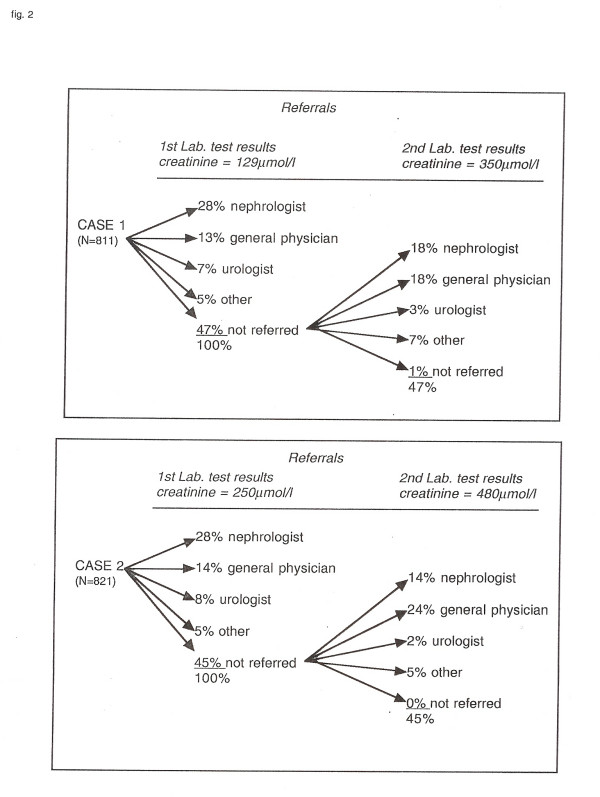
General practice referral routes by creatinine level.

Figure 2 outlines general practitioner referral routes by level of creatinine. While almost all patients were referred at higher levels (350 and 480 μmol/l), referral to a nephrologist decreased in likelihood as the scenarios became more complex, including having higher creatinine levels.

The majority of patients (99%) were referred to a specialist at some point; 46% were referred after the first set of laboratory test results were available. Most were referred to either a nephrologist or a general physician (table 1).

There was no age difference in referral patters with older patients referred as often to a nephrologist as younger patients (chi-square = 0.73), similarly there were no referral rate difference between severe cases and moderate cases (chi-square = 1.30).

Referral levels varied widely with the full range of cases (0–32; median = 15) referred by different general practitioners after first laboratory results were available. Referral rates did not differ by general practitioner according to sex, practice size or current experience with renal patients. One-way analysis of variance indicated a significant interaction between general practitioner training experiences and referral rates; (a) general practitioners who had trained on a renal team (14% of general practitioners), (b) general practitioners who had no specific renal experience in training (60%) and (c) general practitioners who, while not training on a renal team, had some hospital experience with renal patients (26%). Scheffe post hoc comparisons indicated no significant differences between GPs with differing experiences.

## Conclusion

Previous studies have examined reported referral levels to consultants [8,9], but have not focused on differences across general practitioners in their reported referral strategies. Referral across the complete range of cases presented, with no influence of age, marital status or clinical severity, illustrates that no consensus exists about an optimal referral strategy. Such a result is consistent with similar vignette-based research that found substantial variation in dialysis decision making among consultant nephrologists in Northern Ireland [21].

The practical implications of this study can be best understood with regard to, firstly, referral to any specialist and secondly, referral to a nephrologist. The first key issue is whether GPs referred for further investigation. Study findings indicate under-referral of cases for specialist attention by general practitioners in the light of significant renal impairment. Neither general practitioner nor patient characteristics influenced referral. The lack of influence of patient factors is encouraging given evidence that older age and co-morbidity have been inappropriately associated with lower rates of referral [22]. However creatinine levels of 250 μmol/l (case 2) are approaching chronic renal failure levels (≥ 300 μmol/l) [23] yet only 55% of these cases would be referred on to any specialist

The second point of interest concerns whether GPs referred to a nephrologist. Referral to a nephrologist decreased in likelihood as the scenarios became more complex. The imperative to tackle late referral is underlined by estimates that suggest that 25% to 50% of patients worldwide who commence renal replacement therapy are referred late to a nephrology service [23]. Early referral helps to optimize health care use and patient management [15], and enables the identification of patients at risk of rapid deterioration in renal function and/or complications such as anemia, hypertension and cardiovascular disease.

It is worth noting that active management of renal failure by dialysis is a medical innovation of the latter half of the twentieth century. Many of the GPs studied may not have experienced an active approach to dialysis in training. Low numbers of nephrologists available (10 in 1996 in Ireland) may also have influenced referral strategies.

Accepting the limitations of research using 'paper-case' scenarios and reported behaviours, the study did find a pattern of under-referral and/or late-referral for advanced renal failure. This reported practice is consistent with a reported patient referral rate in Ireland. While published guidelines advocate early diagnosis and prompt treatment of renal disease [2], it is not clear at what point direct referral to a nephrologist is the most efficient management strategy for patients with renal difficulties. What is clear from reported practice in this study is that a significant proportion of patients presenting with symptoms of chronic renal failure would not be referred to a specialist. The very divergent management patterns identified across practitioners highlights the need for guidance to general practitioners on appropriate management including referral criteria, referral thresholds and appropriate referral routes. One potential way to promote earlier referral of patients with chronic renal failure is the adoption of the glomerular filtration rate (GFR) as a measurement of kidney function. This new classification of kidney disease, launched by the American Kidney Foundation [24], is based on estimated GFR calculated from serum creatinine, age and sex. This is in many countries now being reported directly to primary care, and is considered a better indicator for detecting poor renal function than is serum creatinine on its own [25]. Ongoing monitoring of general practitioner referral patterns is needed to ensure that such developments translate into appropriate referral from primary care for this serious but manageable medical condition.

## Competing interests

The author(s) declare that they have no competing interests.

## Authors' contributions

AM contributed to the design of the study, collected the data and drafted the manuscript

HMcG designed the study and contributed to the writing of the study

WS and JD developed the standardized case scenarios, and contributed to the writing of the study

## Funding

Irish Kidney Association

## Pre-publication history

The pre-publication history for this paper can be accessed here:



## Figures and Tables

**Table 1:** General practitioner referral patterns for standardised patient cases indicating development of end-stage renal failure

**Patient characteristic**	**Specialist Referral by GP**			
	Nephrologist %	General Physician %	Urologist %	Other %
	
All cases	44	37	7	11*
Younger patient (age 40)	47	36	7	10
Older patient(age 70)	41	39	7	13
Moderate symptoms	40	42	6	12
Severe symptoms	48	32	8	12

*1% of patients not referred to specialists; Moderate symptoms = 129 to 350 μmol/l creatinine, Severe symptoms = 250 to 480 μmol/l creatinine
